# Effect of Levothyroxine on Pregnancy Outcomes in Pregnant Women With Hypothyroxinemia: An Interventional Study

**DOI:** 10.3389/fendo.2022.874975

**Published:** 2022-04-19

**Authors:** Guohua Li, Yang Liu, Xiujuan Su, Shijia Huang, Xiaosong Liu, Qiaoling Du

**Affiliations:** ^1^ Department of Reproductive Immunology, Shanghai First Maternity and Infant Hospital, School of Medicine, Tongji University, Shanghai, China; ^2^ Department of Obstetrics, Shanghai First Maternity and Infant Hospital, School of Medicine, Tongji University, Shanghai, China; ^3^ Clinical Research Center, Shanghai First Maternity and Infant Hospital, School of Medicine, Tongji University, Shanghai, China

**Keywords:** levothyroxine, hypothyroxinemia, hypertensive disorder of pregnancy, maternal outcomes, miscarriage

## Abstract

**Context:**

Adverse maternal outcomes and perinatal complications are associated with maternal hypothyroidism. However, the utility of levothyroxine (L-T4) in the treatment of pregnant women with hypothyroxinemia is unclear.

**Objective:**

This study aimed to evaluate the effects of L-T4 on maternal and perinatal outcomes in pregnant women with hypothyroxinemia.

**Methods:**

The nonrandomized interventional study was conducted at Shanghai First Maternity and Infant Hospital, Punan Hospital of Shanghai, and Beicai Community Health Center of Shanghai. The pregnant women with hypothyroxinemia from the first trimester were enrolled and divided into treatment and control groups. 463 taking L-T4 and 501 not administering L-T4 were analyzed in the study. All participants were screened for TPOAB/TGAB antibody status.

**Main Outcome:**

The primary outcome of the study was the hypertensive disorder of pregnancy (HDP), measured as the proportion of HDP. In addition to this primary outcome, some secondary outcomes will be measured: miscarriage, gestational diabetes mellitus, premature rupture of membranes, placental abruption, intrahepatic cholestasis of pregnancy, fetal distress, macrosomia, and neonates admitted to the neonatal intensive care unit (NICU). The effects of L-T4 on the incidence of adverse pregnancy outcomes and perinatal complications were compared.

**Results:**

Multivariate logistic regression analysis showed that L-T4 treatment (adjusted odds ratio = 1.78 [95% CI = 1.00-3.16], *p* = 0.04) significantly reduced the incidence of miscarriage. Otherwise, lower neonates admitted to the NICU were strongly associated with the L-T4 group (adjusted odds ratio = 1.36 [95% CI = 1.01 – 1.83], p = 0.04). There were no significant differences in the incidence rates of other adverse maternal outcomes and perinatal complications between pregnant women with hypothyroxinemia receiving and those not receiving L-T4 treatment.

**Conclusion:**

The incidence of HDP was not significantly reduced using L-T4 in pregnant women with hypothyroxinemia. The results of this study also showed that L-T4 treatment significantly reduced the miscarriages rate and the proportion of newborns admitted to the NICU.

## Introduction

Hypothyroxinemia is defined as a normal maternal thyroid-stimulating hormone (TSH) concentration in conjunction with a low maternal free thyroxine (FT4) concentration. Thyroid hormones are necessary for embryo growth and development (1). Hypertensive disorder of pregnancy (HDP) is a clinically challenging complication of pregnancy, which accounts for 14% of all maternal deaths (2). HDP includes chronic hypertension, pregnancy-induced hypertension, preeclampsia, chronic hypertension with preeclampsia, and eclampsia. Pregnant women with asymptomatic subclinical hypothyroidism are at risk of severe preeclampsia (3). Buimer et al. reported that women with severe HDP may have lower transient FT4 levels, without evidence of a thyroid disorder (4). Pregnant women with preeclampsia have lower FT4 levels in early pregnancy as well as when they develop preeclampsia compared to normotensive pregnant women (5). The results of a study conducted in Chinese pregnant women showed that FT4 levels were lower in preeclamptic and gestational hypertensive women than in normotensive women (6). Our group also found an increased risk of gestational hypertension in pregnant women with isolated maternal hypothyroxinemia (IMH) (the TPOAb-negative type of hypothyroxinemia (7). Furthermore, we have recently shown that hypothyroxinemia was associated with an increased risk of preeclampsia-eclampsia in women with persistent hypothyroxinemia in the first half of pregnancy (8). Therefore, low FT4 in the first trimester may be associated with the development of HDP. However, there are few studies on the effect of L-T4 treatment on the incidence of HDP. To investigate the role of L-T4 in IMH, Gong et al. conducted a prospective study that included 225 cases of IMH in the first trimester, of which 106 received L-T4 treatment and 95 did not receive L-T4 treatment, and there was no statistically significant difference in the incidence of gestational hypertension or preeclampsia between the two groups (9). However, pregnant women in this study were included from the second trimester and the sample size was small. Moreover, this study did not include a comparative study of antibody-positive patients. Therefore, it is necessary to study the therapeutic effect of L-T4 on both antibody-positive and antibody-negative pregnant women with hypothyroxinemia.

Alternatively, hypothyroxinemia is also a risk factor for some other adverse clinical outcomes. hypothyroxinemia in the first trimester is associated with a higher risk of shortening of the head and hip length of the embryos (10), increased spontaneous abortion (11), preterm birth rate (12), and increased incidence of macrosomia (9, 12). Additionally, lower levels of FT4 during pregnancy are risk factors for gestational diabetes mellitus (GDM) (6, 13). Hypothyroxinemia can adversely affect neurodevelopment in the fetus, with the offspring showing an increased risk of autism (14, 15).

Before conducting this interventional study, we first established reference ranges for FT4 and TSH using 193 healthy pregnant women according to the method recommended by the National Academy of Clinical Biochemistry (16). These pregnant women were independent and not included in our subsequent intervention study. We then recruited women with hypothyroxinemia in the first trimester and intervened to assess the impact of L-T4 treatment on adverse maternal outcomes and perinatal complications. Furthermore, for pregnant women with hypothyroxinemia, with or without TPOAb and/or thyroglobulin antibody (TgAb) as adjustment factors to analyze the effect of L-T4 intervention from the first trimester on intrauterine growth and pregnancy outcomes.

## Methods

### Study Design and Ethics Approval

This nonrandomized interventional study was approved by the Ethics Committee of the Shanghai First Maternity and Infant Hospital, School of Medicine, Tongji University (Trial registration number: ChiCTR1900025560). Participants were recruited from the clinics of Shanghai First Maternity and Infant Hospital, Punan Hospital of Shanghai, and Beicai Community Health Center of Shanghai. Shanghai is in an iodine-sufficient area, and the Shanghai First Maternity and Infant Hospital is a tertiary academic medical center. Written informed consent was obtained from all the eligible participants.

### Diagnosis of Hypothyroxinemia

We established a reference range for the three trimesters according to the National Academy of Clinical Biochemistry (NACB) criteria (16). We measured the levels of TSH and FT4 in 193 pregnant women. The 2.5^th^, 5^th^, 10^th^, 90^th^, 95^th^, and 97.5^th^ percentiles of TSH and FT4 in the three trimesters are shown in **Table S1** of **Supplementary Materials**. The diagnostic criteria for hypothyroxinemia were as follows: TSH range, 2.5^th^ – 97.5^th^ percentile; and FT4 levels in the lower 5^th^ percentile of the reference range described above. Detailed method description was provided in the **Supplementary Material**.

### Recruitment of Participants

The first participant enrollment started on November 1, 2019, and the final follow-up was completed on October 20, 2021, with follow-up till the postnatal period of all the participants. Routine ultrasound and blood tests for thyroid function, vitamins, liver function, lipid profile, fasting plasma glucose, blood count, and other analyses were performed in all pregnant women at the study centers. Pregnant women with hypothyroxinemia were eligible for inclusion in the trial if they met the following criteria: (1) aged 19 - 40years; (2) less than 13 + ^6^ weeks of gestation. Exclusion criteria were as follows: (1) multiple pregnancies; (2) conception by assisted reproductive technology; (3) chronic diseases, such as hypertension, diabetes, and systemic lupus erythematosus; (4) history of thyroid diseases or taking drugs affecting thyroid hormones. All participants answered a questionnaire on demographics. All participants underwent ultrasonography at approximately 7 weeks of gestation, and the duration of pregnancy was calculated based on the date of menstruation and confirmed by ultrasonography.

### Intervention and Follow-Up

Participants were informed about the study by an investigator. At the time of the initial design of this project, it was indeed designed and registered as a randomized controlled study. However, several problems were encountered in the implementation process: first, we did not finally obtain enough samples due to patient wishes and the COVID-19 pandemic, many pregnant women chose to return to their local hospital but not in Shanghai, in addition, some participants wanted to receive the L-T4 because they were fearful of the adverse effect of hypothyroxinemia on pregnancy and some participants don’t bother to attend, so participants were enrolled according to their wishes but not randomized. To reduce the bias, we increased the sample size and controlled the age and gestational age of the subjects.

Participants in the intervention group orally received 25 μg L-T4 once daily, and those in the control group received no treatment. L-T4 was administered immediately after grouping. Participants were seen by the investigators every four weeks. All participants underwent thyroid function tests every 4 weeks until delivery, reporting adverse events and taking L-T4 for the next dose. L-T4 doses were titrated to maintain TSH levels in the 2.5^th^–97.5^th^ percentile and FT4 levels within the 5^th^–97.5^th^ percentile in different trimesters. Moreover, if the TSH level was higher than the 97.5^th^ percentile or FT4 was lower than the 5^th^ percentile, the dose of L-T4 was increased; when TSH level was lower than the 2.5^th^ percentile or FT4 was higher than the 97.5^th^ percentile, L-T4 was discontinued. Interviewers performed prenatal visits every 4 weeks to monitor medication adherence. Pregnancy outcome data were extracted from the electronic medical records. If participants in the control group developed subclinical hypothyroidism, hypothyroidism, or hyperthyroidism, an obstetrician managed them with usual care.

### Statistical Analysis

Continuous variables are presented as mean (standard deviation [SD]), whereas categorical variables are presented as numbers (percentages). The *t*-test was performed to analyze normally distributed data. The chi-square test and Fisher’s exact test were used to analyze categorical variables. The primary outcome was additionally analyzed by binary logistic regression. Independent variables with a p-value of < 0.05 in the univariate analysis were selected for multivariate analysis. Multivariate logistic regression analysis was performed after adjusting for possible confounders to determine significant effects on HDP and calculated as an adjusted odds ratio (95% confidence interval [CI]). The final model retained only significant predictors.

To investigate the effect of L-T4 on secondary outcomes, univariate and multivariate logistic regression analyses were performed to examine the association of independent variables with outcome variables. TPOAB/TGAB positive or not was used as a controlling factor in all the multivariate logistic regression analyses. In addition, age was used as an adjustment factor for miscarriage; age, history of family HBP was used as an adjustment factor for HDP; BMI and history of family HBP were used as an adjustment factor for GDM; GBS positive or not was used as an adjustment factor for premature rupture of membranes (PROM); the uterine scar was used as an adjusted factor for cesarean section.

All statistical analyses were performed using R statistical software v4.0 (package stats). Two-tailed *p*-values < 0.05 were considered statistically significant.

## Results

### Characteristics of Hypothyroxinemia Participants

**Figure 1:**
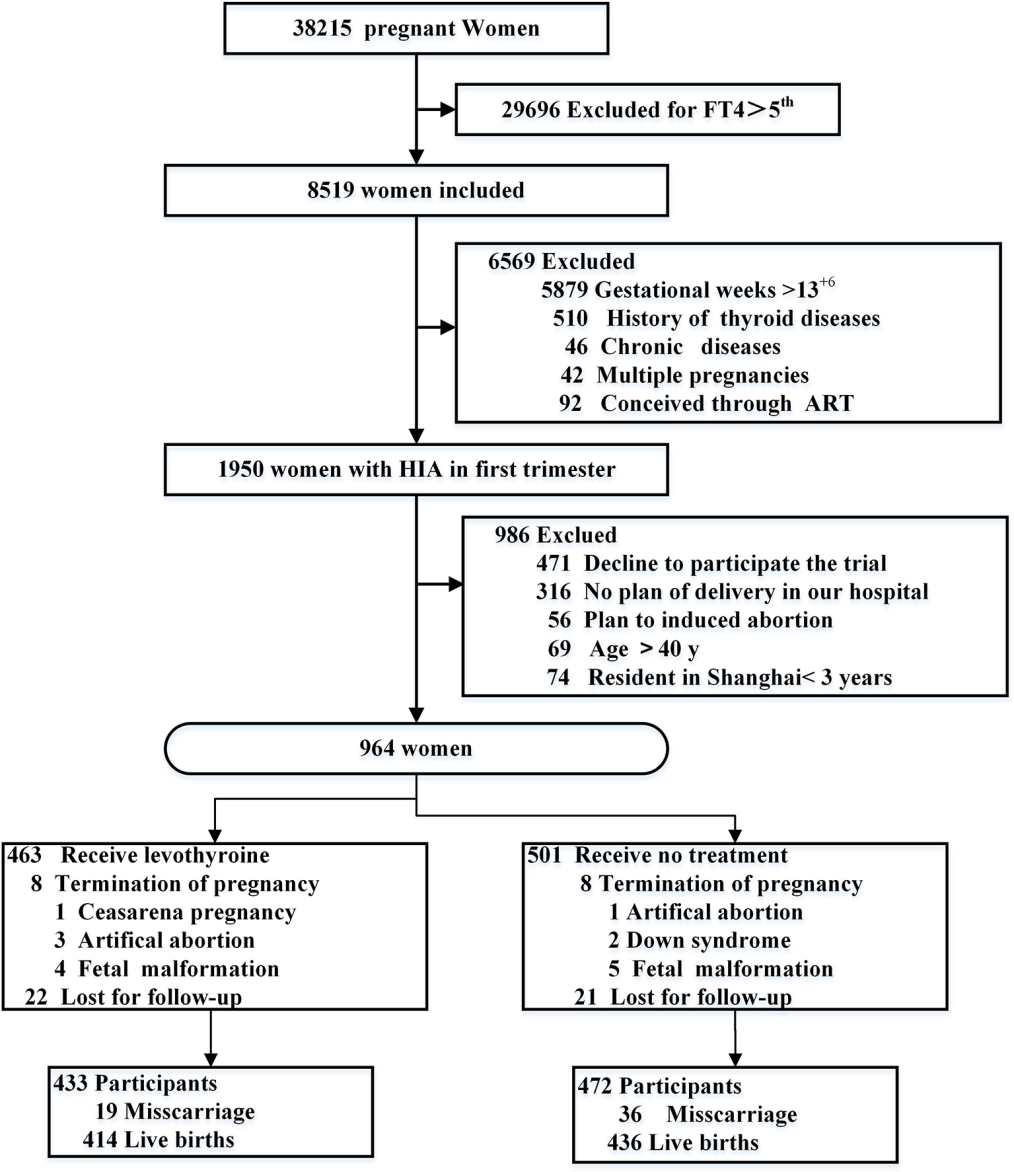
Flowchart of the study population.

A total of 38,215 women were screened, and 964 pregnant women who met the eligibility criteria were included in this study (**Figure 1**). Of these, 463 were assigned to the L-T4 group (intervention group) and 501 to the control group. The gestational weeks of pregnant women in the intervention and control groups were 57.88 days, and 58.78 days (**Table 1**), respectively. The demographic characteristics of the two groups are shown in **Table 1**. There were significant differences in TPOAB/TGAB positive, family history of hypertension, and diastolic pressure at the first prenatal care between the intervention and control groups (*p* < 0.05).

**Table 1:** Demographic characteristics of pregnant women with hypothyroxinemia.

	NoL-T4 N=501	L-T4 N=463	*P* value
Duration of pregnancy at enrollment (day)	57.88 ± 12.20	58.78 ± 10.08	0.22
TPOAB or TGAB	121 (24.2%)	155 (33.5%)	0.01
Age (year)	30.48 ± 3.89	30.31 ± 3.84	0.64
History of term delivery	139 (27.7%)	135 (29.2%)	0.63
History of spontaneous abortion	73 (14.6%)	51 (11.0)	0.10
History of induced abortion	113 (22.6%)	106 (22.9)	0.90
Family history of hypertension	112 (22.4%)	131 (28.3)	0.03
Family history of diabetes	37 (7.4%)	38 (8.2%)	0.63
Systolic pressure at the first prenatal care	105.41 ± 23.02	106 ± 18.25	0.30
Diastolic pressure at the first prenatal care	66.51 ± 15.80	66.77 ± 12.79	0.01
Pre-pregnancy BMI (kg/m^2^)	21.44 ± 2.68	21.60 ± 302	0.78
Uterine scar	64 (12.8%)	59 (12.7%)	0.99

501 subjects did not treat with L-T4 and 463 subjects treated with L-T4 are presented in this table. For continuous variables, mean ± standard deviation (SD) are presented; for categorical variables, absolute numbers and percentage of the total are presented. Systolic and diastolic blood pressure at first prenatal care. TPOAB, thyroid peroxidase antibody; TGAB, thyroglobulin antibody.

### Analysis of Risk Factors of HDP

L-T4 treatment did not reduce the incidence of HDP. Logistic regression was further used to predict factors associated with HDP. Independent variables with *p* < 0.05, in univariate analysis, were selected for multivariate analysis. After controlling for these factors, multivariate logistic regression analysis showed that family history of hypertension (adjusted odds ratio = 2.85 [95% CI = 1.37–6.0], *p* = 0.005) and pre-pregnancy BMI (adjusted odds ratio = 1.23 [95% CI = 1.11–1.37], *p* < 0.001) had significant effects on the incidence of HDP (**Table S2**).

### Analysis of Miscarriages

There were 19 cases of miscarriages in the L-T4 group and 36 cases in the control group. There was a significant difference in the number of miscarriages between the two groups (*p-* value = 0.04). After controlling for TPOAB/TGAB positive or not and age, multivariate logistic regression analysis showed that L-T4 treatment (adjusted odds ratio = 1.78 [95% CI = 1.00–3.16], *p* = 0.04) significantly reduced the incidence of miscarriage (**Table 2**).

**Table 2:** Adverse Pregnancy outcomes among pregnant women with hypothyroxinemia in the levothyroxine (L-T4) treatment group or controls.

	NoL-T4 N=501	L-T4 N=463	Unadjusted			Adjusted model		
	n (%)	n (%)	cOR	95%CI	p value	aOR	95%CI	p value
Miscarriage	36 (7.2)	19 (4.1)	1.81	1.02-3.20	0.04	1.78	1.00-3.16	0.04*
Hypertension disorder of pregnancy	16 (3.2)	24 (5.2)	0.61	0.32-1.15	0.13	0.63	0.32-1.21	0.16
Gestational diabetes mellitus	49 (9.8)	59 (12.7)	0.74	0.50-1.11	0.15	0.76	0.46-1.24	0.27
Premature rupture of membranes	78 (15.6)	82 (17.7)	0.86	0.61-1.20	0.37	0.86	0.61-1.22	0.41
Intrahepatic Cholestasis	6 (1.2)	6 (1.3)	0.92	0.30-2.88	0.89	0.98	0.31-3.09	0.97
Placenta praevia	16 (3.2)	12 (2.6)	1.24	0.58-2.65	0.58	1.20	0.56-2.58	0.64
Placental abruption	3 (0.6)	1 (0.2)	2.78	0.29-26.85	0.38	3.12	0.32-30.43	0.33
Intrauterine growth retardation	6 (1.6)	4 (0.9)	1.39	0.39-4.96	0.61	1.41	0.39-5.05	0.60
Premature delivery	21 (4.2)	20 (4.3)	0.97	0.52-1.81	0.92	0.87	1.06-1.99	0.87
Cesarean section	208 (41.5)	174 (37.6)	0.80	0.61-1.04	0.10	0.75	0.55-1.01	0.06
Breech delivery	17 (3.4)	10 (2.2)	1.59	0.72-3.51	0.25	1.67	0.75-3.69	0.21
Group B streptococcus infection*	22 (4.4)	27 (5.8)	0.74	0.42-1.32	0.31	0.68	0.38-1.22	0.20
Fetal distress*	34 (7.8)	23 (5.6)	1.44	0.83-2.49	0.19	1.40	0.81-2.44	0.23
Macrosomia*	25 (5.7)	33 (8.0)	0.70	0.41-1.20	0.20	0.72	0.42-1.24	0.23
Small for gestation age*	43 (9.9)	34 (8.2)	1.22	0.76-1.96	0.40	1.23	0.77-1.98	0.39
Postpartum hemorrhage*	9 (2.1)	13 (3.1)	0.65	0.28-1.54	0.33	0.71	0.30-1.69	0.43
Apgar score at 1 min ≤ 7*	4 (0.9)	2 (0.5)	1.91	0.35-10.47	0.46	2.39	0.43-13.34	0.32
Neonates admitted to NICU*	148 (33.9)	114 (27.5)	1.35	1.01-1.81	0.04	1.36	1.01-1.83	0.04*
Prematurity^#^	9 (1.8)	14 (3.0)						
Fetal distress^#^	50 (10.0)	21 (4.5)						
Infection suspected or confirmed^#^	75 (15.0)	60 (13.0)						
Other^#^	14 (2.8)	19 (4.1)						

cOR, crude odds ratio; CI, confidence interval; aOR, adjusted odds ratio; NICU, neonatal intensive care unit. *L-T4 n = 414 NoL-T4 n = 436. TPOAB/TGAB positive or not was used as a controlling factor in all the multivariate logistic regression analyses. In addition, age was used as an adjustment factor for miscarriage; age, history of family HBP was used as an adjustment factor for HDP; BMI and history of family HBP were used as an adjustment factor for GDM; GBS positive or not was used as an adjustment factor for premature rupture of membranes (PROM); the uterine scar was used as an adjusted factor for cesarean section. ^#^Reasons for admission to the NICU.

### Comparison of Incidence in Other Adverse Pregnancy Outcomes

The rates of adverse maternal outcomes and perinatal complications, including GDM, PROM, intrahepatic cholestasis, placenta praevia, placental abruption, intrauterine growth retardation, premature delivery, cesarean section, breech delivery, group B streptococcus infection, fetal distress, macrosomia, small for gestation age, postpartum hemorrhage, Apgar score at 1 min ≤7, neonates admitted to neonatal intensive care unit (NICU), were analyzed in the intervention and control groups. Since there were significant differences in the levels of autoantibodies between the intervention and control groups, and thus, we performed logistic regression analysis with TPOAB/TGAB positive or not as controlling factors. Controlling for the effect of TPOAB/TGAB positive or not, lower neonates admitted to the NICU were strongly associated with the L-T4 group (adjusted odds ratio = 1.36 [95% CI = 1.01-1.83], p = 0.04). There was no significant difference in the incidence rate of other adverse pregnancy outcomes between the intervention and control groups (p > 0.05, **Table 2**).

## Discussion

This interventional study showed that controlling for TPOAB/TGAB positivity or not, L-T4 treatment in the first trimester significantly reduced the miscarriage rate of hypothyroxinemia pregnant women and reduced the proportion of newborns admitted to the NICU. There were no significant differences in the incidence of other adverse maternal outcomes and perinatal complications between hypothyroxinemia pregnant women with and without L-T4 treatment. Additionally, as TSH and FT4 levels vary significantly in different populations, an accurate assessment of maternal thyroid function during pregnancy remains difficult; thus, the use of population-based, trimester-specific reference ranges is the best approach to address this problem (17). Therefore, we established a reference for levels of TSH and FT4 in the three trimesters that ensured the reliability of the results of this study.

Gong et al. conducted a prospective study with 225 cases of IMH in the first trimester and found no difference in the occurrence of HDP between women treated with L-T4 (*n* = 106) and those not treated with L-T4 (*n* = 95) (9). The sample size in our study was larger, which allowed us to observe differences in spontaneous abortion and NICU in pregnancy outcomes. Moreover, the pregnant women in our study were included from the first trimester (about 58 days on average) rather than the second trimester. Starting the study in the first trimester was more conducive to our investigation of the effect of L-T4 on spontaneous abortion. Hypothyroidism with clinical symptoms is known to increase the risk of HDP, and several studies have also found low FT4 levels in pregnant women with HDP (3, 4, 6). A previous study by our group found an increased risk of HDP (OR = 2.66; 95% CI: 1.38–5.10) in pregnant women with IMH (7). In this study, we found that L-T4 treatment failed to significantly reduce the incidence of HDP. The possible reason is that L-T4 supplementation alone is not sufficient for improvement, as the causes of HDP development are complex (18).

Normal pregnant women generally have decreased TSH levels and increased FT4 levels due to the effect of human chorionic gonadotropin (hCG) in the first trimester; whereas patients with thyroid disease who are positive for thyroid autoantibodies have mild thyroid dysfunction (19). Thus, levels of FT4 and TSH do not change with hCG in the first trimester (20) and may lead to hypothyroxinemia in these patients with thyroid disease. Hypothyroxinemia in early pregnancy, especially in those with positive thyroid autoantibodies, may persist into the third trimester and adversely affect the mother and child. We also considered the therapeutic effect of LT4 on TPOAb/TgAb-positive and TPOAb/TgAb-negative pregnant women with hypothyroxinemia in the first trimester. We found no significant difference in the incidence of adverse pregnancy outcomes between the intervention and control groups (**Table 2**). A few clinical trials have shown that levothyroxine therapy reduces the incidence of miscarriage and/or preterm birth in TPOAb-positive pregnant women (21, 22). However, subsequent large-scale, multicenter, randomized controlled trials have shown that L-T4 treatment, in comparison to no L-T4 treatment, does not improve maternal and fetal outcomes in pregnant women with TPOAb-positive, subclinical hypothyroidism, or isolated hypothyroidism (23, 24). The findings of the present study are also consistent with the results of two recent systematic reviews of TPOAb-positive women (25, 26). Two randomized controlled trials investigated the effects of screening and treatment of hypothyroxinemia in pregnancy. In a randomized trial (27), antenatal screening and maternal treatment for hypothyroidism did not result in improved cognitive function in children at 3 years of age. Another study (28) that included subclinical hypothyroidism or hypothyroxinemia during pregnancy found no significant differences in pregnancy outcomes or incidence of adverse events. We supplemented these studies in the manuscript and made a discussion. It should be mentioned that the time at a median gestational age of 12.5 weeks was included in the study by John H. Lazaru et al. (27), while 16.7 weeks of gestation in the study by Casey B M et al. was (28). Early thyroid function plays an important role in fetal and pregnancy outcomes. Therefore, it remains unknown whether intervention in the first trimester will have a significant impact on fetal development and pregnancy outcome.

In this study, we found that L-T4 treatment of hypothyroxinemia in early pregnancy can reduce the occurrence of miscarriage. The fetus is completely dependent on maternal thyroid hormone during early pregnancy, a critical period of vulnerability to abortion. Thus, the results of this study suggested that L-T4 supplementation in early pregnancy may be associated with a reduction in the rate of miscarriage. Alternatively, the fetus’s critical neurological development occurs in the first trimester, so L-T4 supplementation in early pregnancy may be responsible for reducing neonatal admission to the NICU. Few studies have been conducted on whether L-T4 can reduce spontaneous abortion in the hypothyroxinemia population, mainly due to the late intervention. In a controlled antenatal thyroid screening study, patients were screened at 12 weeks of gestation, and the study subjects were around 8 weeks of gestation, so the outcome of abortion could be observed (29). Therefore, it may be important for the starting time of the intervention for pregnant women with hypothyroxinemia (30). Our findings further support this notion.

This study has some limitations. First, it was conducted in Shanghai, China, and thus, the results must be extrapolated with caution. Second, this study is only an intervention study, and we did not perform neonatal childhood follow-up. Third, the baseline was different between the two groups, although we performed multivariate analysis to control for the effect of confounding factors. The study also had some advantages. First, we established reference levels for TSH and FT4 in the three trimesters and focused on the therapeutic effect of L-T4 on pregnant women with hypothyroxinemia from the first trimester. Second, we considered patients with positive and negative thyroid autoantibody status. Third, the timing of the intervention in this study was in the first trimester (9 weeks), allowing us to better examine the therapeutic effect of L-T4.

In conclusion, this interventional study found no benefit of L-T4 treatment on pregnancy HDP outcomes in pregnant women with hypothyroxinemia in the first trimester. Administration of L-T4 therapy to pregnant women with hypothyroxinemia can significantly reduce the rate of miscarriage and neonatal admission to the NICU. Our results implied that the intervention time of L-T4 may have a significant impact on treatment outcomes, and further multicenter randomized controlled study is needed to investigate the value of L-T4 treatment in the first trimester.

## Data Availability Statement

The raw data supporting the conclusions of this article will be made available by the authors, without undue reservation.

## Ethics Statement

The studies involving human participants were reviewed and approved by Ethics Committee of the Shanghai First Maternity and Infant Hospital, School of Medicine, Tongji University. The patients/participants provided their written informed consent to participate in this study.

## Author Contributions

QD and GL proposed and designed the study. YL, SH, and XL collected data. XS performed the statistical analysis work, including the sample size calculation. GL and XS analyzed and interpreted data. GL drafted the manuscript. QD reviewed and edited the manuscript. QD provided administrative support and funding acquisition; All authors read, revised, and approved the final draft.

## Funding

Technology Project of the Shanghai Pudong New District Health and Family Planning Commission (PW2019D-9).

## Conflict of Interest

The authors declare that the research was conducted in the absence of any commercial or financial relationships that could be construed as a potential conflict of interest.

## Publisher’s Note

All claims expressed in this article are solely those of the authors and do not necessarily represent those of their affiliated organizations, or those of the publisher, the editors and the reviewers. Any product that may be evaluated in this article, or claim that may be made by its manufacturer, is not guaranteed or endorsed by the publisher.
