# Supplementary material for: Effect of Levothyroxine on Pregnancy Outcomes in Pregnant Women With Hypothyroxinemia: An Interventional Study
Source: Front Endocrinol (Lausanne). 2022 Apr 19;13:874975. doi: 10.3389/fendo.2022.874975 (PMC9062082; doi:10.3389/fendo.2022.874975)
Supplement: Supplementary file 1 [file DataSheet_1.docx]

**Diagnosis of HIA**

Maternal blood samples were collected at three different trimesters and centrifuged (10 min with rethawing cycles at 3000 rpm) to obtain serum. Serum TSH, FT4, TPOAb, and TGAB were measured (ADVIA Centaur XP and kits (Siemens, Munich, Germany)). The intra-assay and inter-assay coefficients of variation were < 4.69 and 6.64% for TSH (levels: 0.008–150 mIU/L), < 3.33 and 4.00% for FT4 (levels: 0.1-12.0 ng/dL), <6.80 and 3.40 % for TPOAb (levels: 28–1300 U/mL), and < 5.80 and 5.70% for TGAB (levels:15-500 U/ml). Both TPOAb and TGAB were considered elevated if levels were ≥ 60 U/mL). We included 193 women with naturally conceived singleton pregnancies who tested negative for both TPOAb and TgAb, had no history of recurrent abortion, clinical diagnosis of chronic or autoimmune disease (diabetes, hypertension, inflammatory bowel disease, tumors, SLE, SS, and others), no personal or family history of thyroid disease, had no visible or palpable goiter.

Other clinical data were obtained from physical medical records or electronic medical record systems. The mean values of FT4 and TSH values in the different trimesters and peak levels of other biochemical tests were compared.

**Table S1. Reference of thyroid-stimulating hormone (TSH) and free thyroxine (FT4) in the three trimesters**

|  | p2.5 | p5 | p10 | p90 | p95 | p97.5 |
| --- | --- | --- | --- | --- | --- | --- |
| TSH **^*^** (mIU/L) | 0.09 | 0.29 | 0.5 | 2.59 | 3.25 | 3.54 |
| TSH§ | 0.2 | 0.51 | 0.74 | 3.57 | 4.1 | 4.32 |
| TSH† | 0.38 | 0.74 | 0.97 | 3.06 | 3.59 | 4.22 |
| FT4 **^*^** (ng/dl) | 10.58 | 11.32 | 12.01 | 16.15 | 16.65 | 18.14 |
| FT4§ | 8.78 | 8.92 | 9.24 | 14 | 15.69 | 15.82 |
| FT4† | 8.85 | 9.02 | 9.44 | 12.66 | 13.32 | 14.38 |

*, § and † represent the first, second, and third trimesters, respectively.

**Table S2. Risk factors for hypertension disorder of pregnancy in logistic regression models**

| Risk factor | Univariate | P value | Multivariate | P value |
| --- | --- | --- | --- | --- |
| Family history of hypertension | 3.23 (1.61-6.54) | < 0.001 | 2.85 (1.37-6.0) | 0.005 |
| Pre-pregnancy body mass index | 1.26 (1.14-1.40) | < 0.001 | 1.23 (1.11-1.37) | < 0.001 |

The data are presented as odds ratio (95% confidence interval).
